# Structural-Functional Correlates of Response to Pedunculopontine Stimulation in a Randomized Clinical Trial for Axial Symptoms of Parkinson’s Disease

**DOI:** 10.3233/JPD-225031

**Published:** 2023-06-13

**Authors:** Sorin Breit, Luka Milosevic, Georgios Naros, Idil Cebi, Daniel Weiss, Alireza Gharabaghi

**Affiliations:** a Department for Neurodegenerative Diseases, Hertie Institute for Clinical Brain Research, and German Centre of Neurodegenerative Diseases (DZNE), University Hospital and University Tübingen, Tübingen, Germany; b Institute for Neuromodulation and Neurotechnology, University Hospital and University of Tübingen, Tübingen, Germany; c Krembil Research Institute, Clinical and Computational Neuroscience, University Health Network, Toronto, Canada

**Keywords:** Deep brain stimulation, Parkinson’s disease, pedunculopontine nucleus

## Abstract

**Background:**

Axial symptoms of Parkinson’s disease (PD) can be debilitating and are often refractory to conventional therapies such as dopamine replacement therapy and deep brain stimulation (DBS) of the subthalamic nuclei (STN).

**Objective:**

Evaluate the efficacy of bilateral DBS of the pedunculopontine nucleus area (PPNa) and investigate structural and physiological correlates of clinical response.

**Methods:**

A randomized, double-blind, cross-over clinical trial was employed to evaluate the efficacy of bilateral PPNa-DBS on axial symptoms. Lead positions and neuronal activity were evaluated with respect to clinical response. Connectomic cortical activation profiles were generated based on the volumes of tissue activated.

**Results:**

PPNa-DBS modestly improved (*p* = 0.057) axial symptoms in the medication-off condition, with greatest positive effects on gait symptoms (*p* = 0.027). Electrode placements towards the anterior commissure (ρ= 0.912; *p* = 0.011) or foramen caecum (ρ= 0.853; *p* = 0.031), near the 50% mark of the ponto-mesencephalic junction, yielded better therapeutic responses. Recording trajectories of patients with better therapeutic responses (i.e., more anterior electrode placements) had neurons with lower firing-rates (*p* = 0.003) and higher burst indexes (*p* = 0.007). Structural connectomic profiles implicated activation of fibers of the posterior parietal lobule which is involved in orienting behavior and locomotion.

**Conclusion:**

Bilateral PPNa-DBS influenced gait symptoms in patients with PD. Anatomical and physiological information may aid in localization of a favorable stimulation target.

## INTRODUCTION

Parkinson’s disease (PD) is generally well-managed by dopamine replacement therapy and/or deep brain stimulation (DBS) of the subthalamic nucleus (STN); however, axial motor symptoms can emerge with disease progression, including postural instability and gait disturbances. These features can result in falls and diminished quality of life, and are often refractory to both dopamine replacement therapy and DBS [1]. Gait is mediated, at least in part, by descending pathways which pass through the brainstem to locomotor-related central pattern generator networks in the spinal cord [2]. The brainstem centers involved are part of the mesencephalic locomotor region (MLR), and include the pedunculopontine nucleus (PPN), and the cuneiform and subcuneiform nuclei. In parkinsonian animal models, the PPN exhibits pathological (cholinergic and non-cholinergic) neuronal cell loss, and altered activity in remaining neurons [3, 4]. Moreover, the PPN demonstrates physiological responses related to motor planning and gait initiation [5]. In animal studies, low-frequency stimulation of the PPN area (PPNa) resulted in spontaneous locomotion, while lesions resulted in gait deficits [5, 6]. It was thus hypothesized that DBS of the PPNa may improve gait and postural symptoms in patients with PD [7]. Herein, we report the outcome of a randomized clinical trial of bilateral PPNa-DBS in patients with PD. We also assessed the impact of electrode position on clinical outcome, the behavior of neurons encountered along surgical trajectories, and explored the connectomic brain network profiles associated with PPNa-DBS.

## METHODS AND MATERIALS

### Patients

Seven patients were treated with bilateral PPNa-DBS within a controlled clinical trial. Patient information is available in Table 1. We included patients in the study whose quality of life was particularly affected not by the symptoms that are usually treated with STN-DBS (tremor, rigidity, akinesia) but by persistent gait and postural symptoms despite optimized medical therapy. Even though these symptoms responded to some degree to medical therapy (Supplementary Table 1), they still relevantly influenced patients’ quality of life to justify DBS surgery. Inclusion criteria were: idiopathic PD according to the British Brain Bank criteria; duration of illness >5 years; age >25 years but <80 years; anti-parkinsonian medication stable for at least one month before surgery; UPDRS ≥30 (med-OFF); Axial-Score ≥16 (med-OFF; composite score of gait and postural symptoms items from UPDRS, defined below in “Outcome Measures”); written declaration of consent. Exclusion criteria included: major depression (Beck Depression Inventory (BDI)>25); cognitive limitations (Mini-Mental-Status-Test (MMST)<25); acute psychosis; surgical contraindications; severe mental illness; serious internal disease (e.g., immunodeficiency, non-curative treated malignant diseases); severe neurological disorders (e.g., epilepsy, brain surgery, traumatic brain injury, brain infarct); previous treatment with DBS or therapeutic basal ganglia lesion; lack of consent or existing support; lack of ability to understand the purpose of the study and the process; participation in other clinical studies; pregnancy. Each patient provided written informed consent prior to participation in the study, and the study was approved by the ethics committee of the University Hospital Tübingen (188/2009MPG1) and was registered at the Regional Administrative Council with the clinical trial registration number DE/CA48/54.4-17/5552.21-1.17/0009692//10/26/2009.

**Table 1:** Patient information

	Sex	Age	Disease	LEDD (mg/d)			Stimulation parameters
		Range	Duration	Preoperative	Stim-OFF	Stim-ON
PPN1	M	60–65	18	2746	1846	1766	L: 1-2+, 2.5V, 60μs, 20 Hz R: 9-10+, 4.0V, 60μs, 20 Hz
PPN2	M	60–65	15	2756	2756	2756	L: 3-2+, 1.7V, 60μs, 20 Hz R: 11-10+, 2.5V, 60μs, 20 Hz
PPN3	F	60–65	12	432	665	623	L: 0-1+, 3.0V, 60μs, 10 Hz R: 8-9+, 3.0V, 60μs, 10 Hz
PPN4	M	65–70	13	1879	1678	1678	L: 0-1+, 2.5V, 60μs, 20 Hz R: 8-9+, 2.5V, 60μs, 20 Hz
PPN5	M	60–65	14	823	642	642	L: 0-1+, 2.8V, 60μs, 10 Hz R: 8-9+, 3.0V, 60μs, 10 Hz
PPN6	F	70–75	22	1486	1453	1453	L: 0-1+, 3.5V, 60μs, 20 Hz R: 8-9+, 3.5V, 60μs, 20 Hz
PPN7	F	60–65	21	1626	1286	1286	L: 3-2+, 2.0V, 60μs, 20 Hz R: 11-10+, 2.0V, 60μs, 20 Hz
average	42.9% F	65.3±3.3	16.4±4.0	1678.3±881.9	1475.1±731.0	1457.7±732.7	–

### Surgical procedures

Bilateral electrode implantations (3389 Model, Medtronic, MN, USA) were performed under local anesthesia after overnight withdrawal from anti-parkinsonian medication. The PPNa was targeted via direct localization using a proton-density MRI protocol at 1.5T [8]. Microelectrode recordings were performed in five of the seven patients, beginning ∼10 mm above the planned target location to ∼5 mm below (30-s recordings at 0.5 mm intervals). An implantable pulse generator (Activa PC, Medtronic, MN, USA) was implanted under general anesthesia in the subclavicular region of each patient.

### Therapy and stimulation parameters

The optimal stimulation parameters (Table 1) were determined individually on the basis of the predominant axial symptom of each patient while avoiding acute/reversible side effects. Initial stimulation parameters (20 Hz, 60μs) were chosen based on the literature, with titration of stimulation intensity. If suitable side effect thresholds were not achieved, stimulation frequency was adjusted to 10 Hz to remain below the side effect threshold and allow for blinded conditions. This titration was done over several days in the course of an inpatient stay in hospital, each session could take up to three hours. All patients received bipolar stimulation to allow for more precise targeting of the electrical field in the mesencephalic brain area. Five patients were stimulated with 20 Hz and two patients with 10 Hz. A stimulation pulse-width of 60μs was used for all patients, and an average stimulation amplitude of 2.75±0.64 V was used.

### Complications and side effects

MRI images acquired postoperatively showed no abnormalities related to asymptomatic bleeding or ischemia. The following postoperative complications were documented: transient motor aphasia without a morphological MRI correlate and without indications for seizure-typical activity in EEG (*n* = 1); erosion of skin and subsequent local infection in the area of the impulse generator at three months postoperatively, with regression after antibiotic therapy (*n* = 1); radius-fracture after fall during Stim-ON (*n* = 1); postoperative dysarthria with regression after several weeks prior to randomization (*n* = 1). Acute, reversible side effects of supra-threshold stimulation included sensory disturbances and oscillopsia; likely explained by spread of the stimulation to neighboring regions [9]. No additional side effects were observed. Psychoses were not documented. Indications of suicidal tendencies did not occur. The BDI scores showed some worsening during Stim-ON, though not statistically significant.

### Study design

**Fig. 1:**
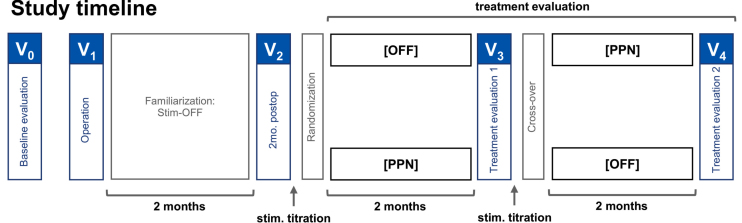
Randomized double-blind cross-over study design. The patients were brought in for baseline evaluations preoperatively. The operation was followed by a two-month familiarization phase with stimulation OFF, and to account for possible micro-lesions effects. At two months postoperatively, the patients visited for a postoperative evaluation, randomization of stimulation settings, and titration of the stimulation settings. At four months postoperatively, the patients visited again for the first treatment evaluation, cross-over, and stimulation titration. The second treatment evaluation, marking the end of the study, was performed at six months postoperatively.

A prospective, randomized, double-blind, cross-over study design was applied with two 2-month treatment periods subsequent to a 2-month postoperative period (see Fig. 1 for detailed study outline). The study compared clinical scores between activated bilateral PPNa-DBS (Stim-ON) and the stimulation OFF condition (Stim-OFF). Surgeries and treatment evaluations were performed within an overall study period of 36 months. After participation in the study, stimulation was activated for all patients and treatment was continued unblinded.

### Outcome measures

Per the registered study protocol, the primary outcome was the “Axial-Score” (UPDRS items 13–15; falling unrelated to freezing, freezing when walking, walking; and 27–31; arising from chair, posture, gait, postural stability, body bradykinesia) comparison between Stim-ON and Stim-OFF, in the medication-OFF condition. Axial-Score subscores were also assessed, including Gait-Score (items 13, 14, 15, 29) and Posture-Score (items 27, 28, 30, 31) as posthoc variables (outside of protocol). Secondary outcome measures included UPDRS-I (Mentation, Behavior, and Mood), II (Activities of Daily Living), and IV (Complications of Therapy) in the medication-OFF condition, and UPDRS-III (Motor Examination) in medication-OFF and medication-ON. Additional secondary outcomes included Schwab & England activities of daily living, Hoehn & Yahr Scale, Freezing of Gait Questionnaire (FOGQ), BDI, MMST, Medical Outcomes Study (MOS) Sleep-Scale, and Parkinson’s Disease Questionnaire (PDQ-39). The Axial-Score (medication-ON), Gait-Score (medication-ON), Posture-Score (medication-ON), UPDRS-II (medication-ON), and Gait and Falls Questionnaire (GFQ) scores were acquired and analyzed as additional variables. The study protocol that specified the applied assessments was submitted in 2009. Therefore, an old UPDRS version was applied in subsequent years to remain consistent throughout the study period. Notably, assessments that are conducted by means of a rater or patient self-report only (and not with objective gait analysis measures) may limit the generalizability of the results.

### Statistical analyses

*Clinical outcome data*: For trial outcome data, 2-tailed Wilcoxon singed-rank tests were reported along with Cohen’s d effect sizes. In addition to Stim-ON versus Stim-OFF comparisons, the Stim-ON condition was compared to preoperative baseline assessments (considered outside of the study protocol). Results of Shaprio-Wilks tests for normality are available in Supplementary Table 2. *Lead placements*: For MRI-based structural correlates, normalized distances (left/right averaged) of the active contacts midpoints were calculated with respect to AC-PC and PMJ [10, 11] lines. Spearman ranked correlations were obtained between Axial-Score improvements (medication-OFF) and proximity to AC-PC and PMJ landmarks (*n* = 6). *Single-neuron activity*: For single-unit analyses, template-matching was done in Spike2 8.10 (Cambridge Electronic Design Ltd., UK). Firing rates were measured and burst indexes (mean divided by mode insterspike interval) were calculated and correlated with one another (*n* = 35; three outliers; Pearson’s correlation). K-means cluster analysis was executed in MATLAB 2018b (MathWorks Inc, MA, USA). Firing rates and burst indexes were compared between clusters and between two patients with more anterior electrode placements and two patients with more posterior electrode placements (2-tailed Mann-Whitney tests). One patient was omitted from structural and single-unit analyses as postoperative MRI images revealed that the active contacts were beyond one standard deviation of the group mean, microelectrode recordings revealed sparse single-unit activity, and the Axial-Score worsened by 1 point. Statistical analyses were performed in SPSS v23.0 (IBM Corp, NY, USA).

**Table 2:** Primary and secondary clinical outcome results

	Scores (mean±standard deviation)			Wilcoxon signed-rank test (p)	
				for double-blind evaluations:	
	Baseline	Stim-OFF	Stim-ON	Stim-ON versus	Stim-ON versus
				Stim-OFF	baseline
Primary endpoint (per protocol)
Axial-Score (med-OFF)	22.0±4.2	21.7±3.2	20.3±4.2	0.057	0.023^*^ (1.349)
Primary endpoint subscores (outside protocol)
Gait-Score (med-OFF)	10.4±1.9	11.0±1.3	8.7±2.1	0.027^*^ (1.678)	0.033^*^ (1.249)
Posture-Score (med-OFF)	11.6±2.7	10.7±2.1	11.6±2.5	0.063 neg.	>0.99
Secondary endpoints (per protocol)
UPDRS-I	3.3±1.5	4.6±2.4	3.1±1.9	0.071	0.854
UPDRS-II	19.1±8.1	20.1±7.9	18.0±7.0	0.461	0.609
UPDRS-III (med OFF)	51.4±14.3	51.0±9.3	46.4±13.1	0.074	0.204
UPDRS-III (med ON)	30.0±10.9	36.0±12.9	32.1±11.9	0.249	0.612
UPDRS-IV	9.6±3.6	7.0±3.1	7.3±2.1	0.798	0.062
Schwab & England	57±14%	57±16%	60±15%	0.157	0.157
Hoehn & Yahr	4.0±0.6	4.0±0.6	3.9±0.4	0.317	0.317
FOGQ	19.0±2.8	19.6±2.4	18.1±4.0	0.288	0.496
BDI	10.7±5.9	10.9±8.0	14.3±8.3	0.072 neg.	0.248
MMST	27.6±3.3	26.3±4.6	27.7±3.4	0.141	0.180
MOS Sleep Scale	49.3±1.8	49.4±5.9	49.0±6.4	0.917	>0.99
PDQ-39 (*n* = 5)
Mobility	25.4±11.9	27.2±12.6	23.4±14.7	0.074	0.336
Activities of daily living	10.6±7.8	13.4±7.4	11.2±6.6	0.197	0.785
Emotional well-being	9.0±5.1	7.0±4.0	9.2±5.3	0.180	0.492
Stigma	4.6±4.3	4.0±3.1	5.0±4.5	0.257	0.581
Social support	3.2±2.5	3.0±3.1	5.0±3.3	0.273	0.059
Cognition	3.2±2.6	3.2±2.2	3.4±1.8	0.854	0.892
Communication	4.0±4.4	4.6±3.1	4.8±1.6	0.713	0.588
Bodily discomfort	4.4±4.3	6.0±7.0	3.6±3.2	0.465	0.496
Additional scores (outside protocol)
Axial-Score (med-ON)	15.6±6.0	16.0±6.5	15.7±2.5	0.865	0.786
Gait-Score (med-ON)	8.0±2.6	7.7±2.9	7.0±1.9	0.343	0.149
Posture-Score (med-ON)	7.6±3.9	8.3±4.2	8.7±2.1	0.595	0.345
UPDRS-II				
(med-ON)	19.1±8.1	20.1±8.0	18.0±7.0	0.461	0.609
GFQ	39.2±9.3	40.6±9.7	33.7±13.8	0.034^*^ (1.256)	0.172

^*^p < 0.05; neg., negative gradient, i.e., worsening; Cohen’s dz effect sizes are parenthesized; PPN3 and PPN6 excluded from PDQ-39 as questionnaires were not completed; GFQ baseline data are also incomplete for PPN3.

### Structural and functional connectomic profiles

In additional to structural imaging analyses in stereotactic space, DBS lead localizations were performed in Lead-DBS, as previously described in detail [12]. Briefly, atlas segmentations were defined by the PPN histological atlas [13], and DISTAL atlas for three STN-DBS comparator patients. The volume of tissue activated (VTA) was calculated for each patient using a finite element approach in Lead-DBS, and the groupwise VTA was used as a seedpoint to create structural (dMRI-based) and functional (fMRI-based) connectomic profiles, estimated from a publicly available PD group connectome. Individual structural and functional cortical activation profiles are also available in Supplementary Figure 1. It should be noted that no correlations were found between PPN VTA overlap and clinical outcome, nor between structural (i.e., fiber count) or functional (i.e., fMRI correlates) connectomic cortical activation profiles and clinical outcome with superior parietal regions of interest.

## RESULTS

### Trial outcomes

Complete results including primary and secondary outcome measures and additional findings are available in Table 1. Notably, the Axial-Score was better with Stim-ON than Stim-OFF (20.3±4.2 versus 21.7±3.2; *p* = 0.057; though not statistically significant at α= 0.05), as was Gait-Score (8.7±2.1 versus 11.0±1.3; *p* = 0.027), while Posture-Score was worse (11.6±2.5 versus 10.7±2.1; *p* = 0.063; not significant at α= 0.05). Additionally, the UPDRS-III medication-OFF (46.4±13.1 versus 51.0±9.3; *p* = 0.074), UPDRS-I (3.1±1.9 versus 4.6±2.4; *p* = 0.071), and Mobility item of PDQ-39 (23.4±14.7 versus 27.2±12.6; *p* = 0.074) scores were all better with Stim-ON compared to Stim-OFF, though not significant at α= 0.05. GFQ scores were also better with Stim-ON compared to Stim-OFF (33.7±13.8 versus 40.6±9.7; *p* = 0.034). Stim-ON versus baseline results are also summarized in Table 1; notable improvements included Axial-Score (20.3±4.2 versus 22.0±4.2; *p* = 0.023) and Gait-Score (8.7±2.1 versus 10.4±1.9; *p* = 0.033).

### Lead placements

**Fig. 2:**
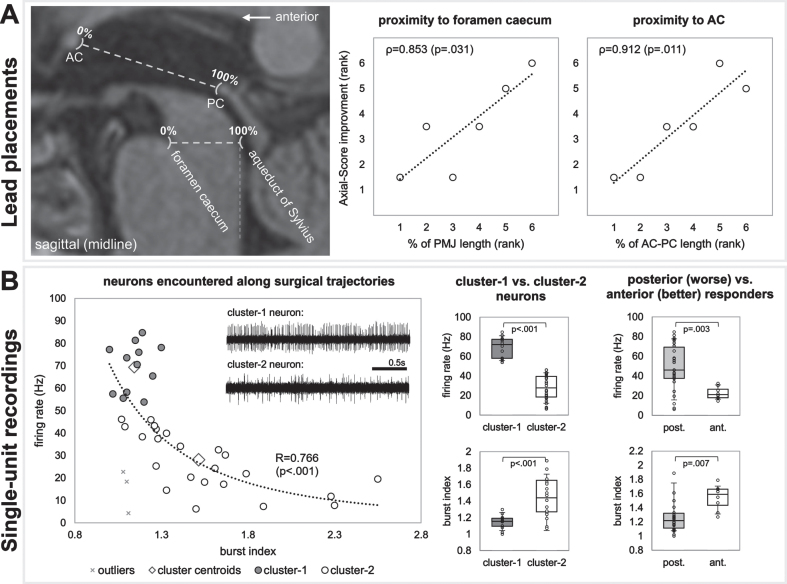
A) Lead placement correlates, and B) single-unit recordings. A) MRI results suggest that more anterior lead placements were favorable. B) Single-unit recordings show two clusters of neurons (cluster-1 being faster and more regular versus cluster-2 being slower and less regular). Neurons resembling cluster-2 were more prominent in recording trajectories of patients with more anterior electrode placements (who had better therapeutic responses).

Based on MRI analyses in stereotactic space, more anterior lead placements appeared to be favorable (Fig. 2A). Ranked correlations were found between Axial-Score improvement and proximity to the foramen caecum (PMJ line; ρ= 0.853; *p* = 0.031), and the AC (AC-PC line; ρ= 0.912; *p* = 0.011). Correlations regarding laterality and superior/inferior distances from PMJ and AC-PC lines were *p* > 0.2.

### Microelectrode recordings

A correlation fit with a power function (*p* < 0.001) was found between neuronal firing rate and burst index (Fig. 2B). Of the two clusters of neurons, cluster-1 were faster (69.3±10.9 Hz versus 27.2±13.2 Hz; *p* < 0.001) with more regular firing patterns (1.14±0.09 versus 1.55±0.39 burst index; *p* < 0.001). Patients with more anterior electrodes (better therapeutic response) had neurons with lower firing rates (22.5±6.37 Hz versus 48.9±23.1; *p* = 0.003) and higher burst indexes (1.54±0.18 versus 1.33±0.37; *p* = 0.007), resembling cluster-2 neurons.

### Structural and functional connectomic profiles

**Fig. 3:**
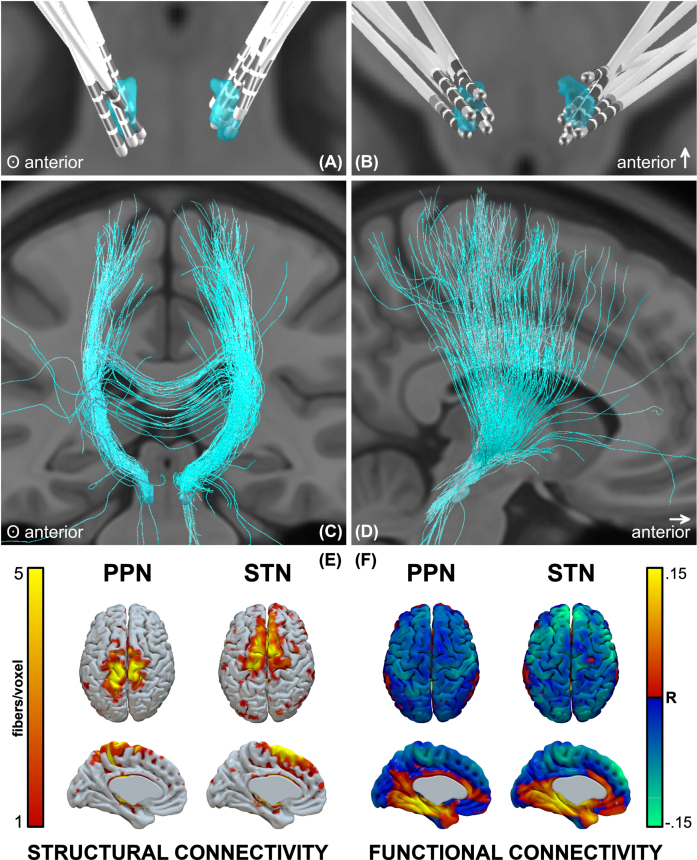
Lead locations and groupwise structural and functional connectomic profiles. A, B) Reconstructions of all electrodes, C, D) fibers traversing the groupwise VTA, and E) structural and F) functional connectivity profiles.

PPNa-DBS leads were localized in Lead-DBS (Fig. 3A, B). The structural connectivity profile showed that the densest projections were in proximity to the midline, spanning the superior parietal lobule, S1, and M1 areas (Fig. 3C–E), whereas the STN profile spanned M1, SMA, and prefrontal regions. The functional connectivity profile showed inverse correlation with midline sensorimotor regions, and to a lesser degree more distal sensorimotor and prefrontal areas (Fig. 3F). The strongest inverse correlations for STN were in prefrontal regions, but also included sensorimotor areas.

## DISCUSSION

Bilateral PPNa-DBS modestly influenced axial features (particularly gait) in the medication-OFF condition. Only two other randomized clinical studies evaluated the efficacy of bilateral PPN-DBS without concurrent STN-DBS [14, 15]. In one study [14], no significant changes were found for the primary outcome measures (Rating Score for Gait Evaluation (RSGE), UPDRS-II, and an ‘axial’ subscore; *n* = 4), though the authors reported reductions in falling (RSGE-6) and FOG (RSGE-7) in three of four patients, as well as improved anticipatory postural adjustments and double-stance durations. In the other study [15], PPN-DBS was compared to DBS of the cuneiform (CuN) nuclei and sham in a randomized double-blind cross-over trial. Two months of PPN-DBS or CuN-DBS did not improve gait and balance disorders. This negative finding remained unchanged when evaluating the same patients two years after surgery [16].

The results of an open-label study (*n* = 5) [17] suggested that bilateral PPNa-DBS was more efficacious than unilateral, and led to improvements in GFQ scores (replicated in a subsequent double-blind study; *n* = 7 [10]) and UPDRS ‘axial’ items (medication-OFF).

Other double-blind studies which applied different stimulation approaches have also reported mixed outcomes. Stefani and colleagues [7] (*n* = 6) reported promising results with combined STN-PPNa-DBS compared to STN-DBS alone; however, these results were not replicated by Ferraye and colleagues [18] (*n* = 6) during double-blind assessments (improvements to FOG and falls were found in open-label assessments). In a study assessing unilateral PPNa-DBS (*n* = 6), Moro et al. [19] did not find improvements in UPDRS-III scores during double-blind evaluations; however, they reported reductions in the UPDRS-II fall item during open-label assessments. In a double-blind follow-up study [20], the authors reported improvements in only the UPDRS-II fall item at 2-years postoperatively (*n* = 8), but no differences in any measures at 4-years (*n* = 6); perhaps the result of disease progression. These equivocal observations were also reported in more recent open-label studies (*n* = 6 [21] and *n* = 5 [22]).

### Lead placements

The finding that more anterior electrode placements were favorable seemed contradictory to that of Goetz and colleagues [11]. However, the electrodes of “bad responders” in the aforementioned study were substantially more anterior than any of the electrodes presented here. Corroborating the findings of Goetz and colleagues with our own, by overlapping the favorable electrode locations in both studies, suggests that a favorable position may exist near the 50% mark of the PMJ line (substantiated by anatomical and imaging studies localizing PPN [8, 9]). Electrode placements too anterior (as in [11]) or too posterior (as shown here) may yield less favorable results. The trajectories in our study were targeted somewhat posteriorly due to an emerging notion that (co-)stimulation of the cuneiform nucleus may yield a positive effects on gait [2, 23]. Although more anterior electrode positions were favorable when assessing electrode position in stereotactic space, analyses which assessed VTA overlap with the PPN in MNI-space did not result in correlations with clinical outcome.

### Single-neuron activity

Assessment of neuronal activity along surgical trajectories suggested that it may be more favorable to target a slower (22.5±6.37 Hz), less regular neuronal population. This observation is in line with a recent report on two cases where similar discharge rates have been detected (19.1±15.1 Hz) [24]. These findings may implicate a particular population of neurons with physiological relevance for influencing gait and may aid in the intraoperative localization of favorable electrode placements. Intraoperative microelectrode recordings alone are insufficient in discerning neuronal subtypes (cholinergic, glutamatergic, GABAergic; all present in PPN); however, speculating on the basis of *in vitro* animal studies and previous *in vivo* human studies suggests that the neurons with slow and irregular firing characteristics and broad spike shapes may be cholinergic [25, 26]. Indeed, cholinergic neuronal cell loss has been described in parkinsonian animal models [4, 5] and pharmacogenetic stimulation of this neuronal population was shown to reverse gait and postural abnormalities [27].

### Structural and functional connectomic profiles

The PPN has connections with the cerebral cortex, multiple basal ganglia and limbic areas, the thalamus, other brainstem regions, the spinal cord, and the cerebellum. These connections implicate the PPN in a variety of functions including movement, cognition, and sleep. With respect to cortical connectivity, dense projections exist between upper extremity regions of the motor cortex, followed by the lower extremity, trunk, and orofacial regions [28]; as are shown here. Overall, the PPN receives direct cortical afferent input from the M1 and S1, and presupplementary and premotor cortices, as well as frontal eye fields.

In adult cats, skilled locomotor performance was disrupted by lesions to motor cortical areas, including M1, S1, and parietal cortices [29]. Direct connections between the PPN and superior parietal lobule have not been previously described; however, these connections (shown here) may be the result of the activation of neighboring MLR regions. The posterior parietal cortex has been implicated visuomotor coordination for gait adjustment upon encountering an obstacle. In cats, when the posterior parietal cortex was bilaterally removed, the hindlimbs did not step over obstacles (even after the forelimbs did) as the obstacle left the visual field [30]. It was thus postulated that the role of the posterior parietal cortex in gait is to register and store the temporospatial relationship between one’s body and prospective obstacles in the short-term memory, in order to produce motor programs for modification of limb trajectories. This may reflect the phenomenon in patients with PD in which gait deficits can be improved by engagement of visual attention and allocation of gait to the working memory [31]. Overall, the cortical regions identified by connectomic profiles of PPNa-DBS VTAs are seemingly in congruence with previously described structural and physiological substrates of gait; however, structural and functional activation profiles did not correlate with clinical outcome.

### Limitations

The medication-OFF Axial-Score and Gait-Score improvements of 7.7% and 16.3%, respectively, were modest; thus, the results should be interpreted with caution. However, a recent study demonstrated that axial symptom score worsening (the only predictor of mortality in patients with PD) was 27% over the course of 10 years [32]; thus, any attenuation of axial symptoms may be meaningful. It is important to note that posthoc assessments in the medication-ON condition did not result in these changes even though the patients reported a significant improvement in the Gait and Freezing Questionnaire. This discrepancy between clinical scores and patient self-assessment needs to be considered in future studies. Two other limitations of this study are the lack of long-term follow-up, as well lack of improvements to PDQ-39 scores. Moreover, with regards to the primary outcome, the dopamine response alone was greater than the stimulation response (Supplementary Table 1). Moreover, due to the low sample counts for structural and functional analyses, the generalizability of these findings remains to be established. Indeed, this and other PPNa-DBS trials have been limited by small sample sizes; the largest of which has been seven [1, 7, 10, 14, 19, 20]. With such low sample sizes, robust effects are necessary to achieve reasonable statistical power, placing a great importance on the selection of appropriate/meaningful clinical outcome measures. Beyond that, most studies have been limited by a lack of additional quantitative symptomatic assessments, including our own. While the initial interest in targeting PPN for gait related symptoms stemmed from early basic research showing the involvement of PPN in locomotion and parkinsonism [3, 5, 6, 27], some of this literature has not been consistently replicated [33, 34]. Considering this and the lack of consistent results within controlled trials, the justification and clinical application of PPNa-DBS have been reconsidered [35].

### Conclusions

Bilateral PPNa-DBS modestly influenced axial symptoms, particularly with respect to gait, in the medication-OFF condition. Electrode placements near the 50% mark of the PMJ line, where slow irregular neurons were encountered along surgical trajectories, yielded better therapeutic responses.

## Supplementary Material

Supplementary Material

## Data Availability

The data will be available upon request, as the ethics approval does not allow uploading the datasets to a publicly available repository.
