# Supplementary material for: Structural-Functional Correlates of Response to Pedunculopontine Stimulation in a Randomized Clinical Trial for Axial Symptoms of Parkinson’s Disease
Source: J Parkinsons Dis. 2023 Jun 13;13(4):563–73. doi: 10.3233/JPD-225031 (PMC10357146; doi:10.3233/JPD-225031)
Supplement: Supplementary Material [file jpd-13-jpd225031-s001.pdf]

Supplementary Material

Structural-Functional Correlates of Response to Pedunculopontine Stimulation in a Randomized Clinical Trial for Axial Symptoms of Parkinson's Disease

Supplementary Table 1. Preoperative improvements to primary outcome score and subscores with levodopa.

	Axial-Score improvement	Gait-Score improvement	Posture-Score improvement
PPN1	13 (59.1%)	7 (63.6%)	6 (54.5%)
PPN2	4 (13.3%)	1 (7.1%)	3 (18.9%)
PPN3	8 (36.4%)	2 (20.0%)	6 (50.0%)
PPN4	7 (43.8%)	1 (12.5%)	6 (75.0%)
PPN5	6 (28.6%)	3 (27.3%)	3 (30.0%)
PPN6	4 (20.0%)	2 (20.0%)	2 (20.0%)
PPN7	3 (13.0)	1 (11.1%)	2 (14.3%)
average	6.4±3.4 (30.6±17.1%)	2.4±2.1 (23.1±19.1%)	4.0±1.9 (37.5±22.7%)

Values represent Med-OFF minus Med-ON baseline scores; parenthesized values are the % improvement

Supplementary Table 2. Normality of sampling distributions

variable	condition	Shapiro-Wilks (p)	
		individual	paired difference between Stim-ON & baseline / Stim-OFF
Axial-Score (med-OFF)	baseline	0.338	**0.001**
	Stim-OFF	0.087	0.432
	Stim-ON	0.292	-
Gait-Score (med-OFF)	baseline	0.523	0.099
	Stim-OFF	0.819	0.877
	Stim-ON	0.686	-
Posture-Score (med-OFF)	baseline	0.822	**0.024**
	Stim-OFF	0.744	0.062
	Stim-ON	0.866	-
UPDRS-I	baseline	0.126	0.689
	Stim-OFF	0.428	0.523
	Stim-ON	0.271	-
UPDRS-II	baseline	0.927	0.644
	Stim-OFF	0.689	0.809
	Stim-ON	0.813	-
UPDRS-III (med OFF)	baseline	**0.008**	0.217
	Stim-OFF	0.742	0.413
	Stim-ON	0.546	-
UPDRS-III (med ON)	baseline	0.518	0.389
	Stim-OFF	0.885	0.799
	Stim-ON	0.859	-
UPDRS-IV	baseline	0.999	0.433
	Stim-OFF	0.152	0.883
	Stim-ON	0.342	-
Schwab & England	baseline	0.456	**0.000**
	Stim-OFF	**0.013**	**0.000**
	Stim-ON	0.310	-
Hoehn & Yahr	baseline	**0.024**	**0.000**
	Stim-OFF	**0.000**	**0.000**
	Stim-ON	**0.024**	-
FOGQ	baseline	0.178	0.174
	Stim-OFF	0.202	0.167
	Stim-ON	0.426	-
BDI	baseline	**0.042**	0.711
	Stim-OFF	0.541	0.563
	Stim-ON	0.922	-
MMST	baseline	**0.031**	**0.007**
	Stim-OFF	0.114	0.566
	Stim-ON	**0.030**	-
MOS Sleep Scale	baseline	0.292	0.632

	Stim-OFF	0.738	0.529
	Stim-ON	0.381	-
Mobility	baseline	0.886	0.082
	Stim-OFF	0.169	0.171
	Stim-ON	0.063	-
Activities of daily living	baseline	0.985	0.281
	Stim-OFF	0.530	0.332
	Stim-ON	0.478	-
Emotional well-being	baseline	0.497	0.135
	Stim-OFF	0.731	**0.007**
	Stim-ON	0.829	-
Stigma	baseline	0.556	0.254
	Stim-OFF	0.967	0.435
	Stim-ON	0.332	-
Social support	baseline	0.384	**0.009**
	Stim-OFF	**0.037**	0.642
	Stim-ON	0.182	-
Cognition	baseline	0.501	0.314
	Stim-OFF	0.254	0.928
	Stim-ON	0.272	-
Communication	baseline	0.417	0.544
	Stim-OFF	0.065	0.223
	Stim-ON	0.490	-
Bodily discomfort	baseline	0.145	0.823
	Stim-OFF	0.265	0.110
	Stim-ON	0.794	-
Axial-Score (med-ON)	baseline	0.528	0.700
	Stim-OFF	0.859	0.419
	Stim-ON	0.827	-
Gait-Score (med-ON)	baseline	0.083	**0.013**
	Stim-OFF	0.173	**0.010**
	Stim-ON	**0.001**	-
Posture-Score (med-ON)	baseline	0.777	0.789
	Stim-OFF	0.254	0.287
	Stim-ON	0.122	-
UPDRS-II (med-ON)	baseline	0.217	0.177
	Stim-OFF	0.865	0.215
	Stim-ON	0.139	-
GFQ	baseline	0.070	0.777
	Stim-OFF	0.511	0.178
	Stim-ON	0.959	-

bolded values = assumption of normality is not likely to be satisfied (i.e., $p<0.05$)

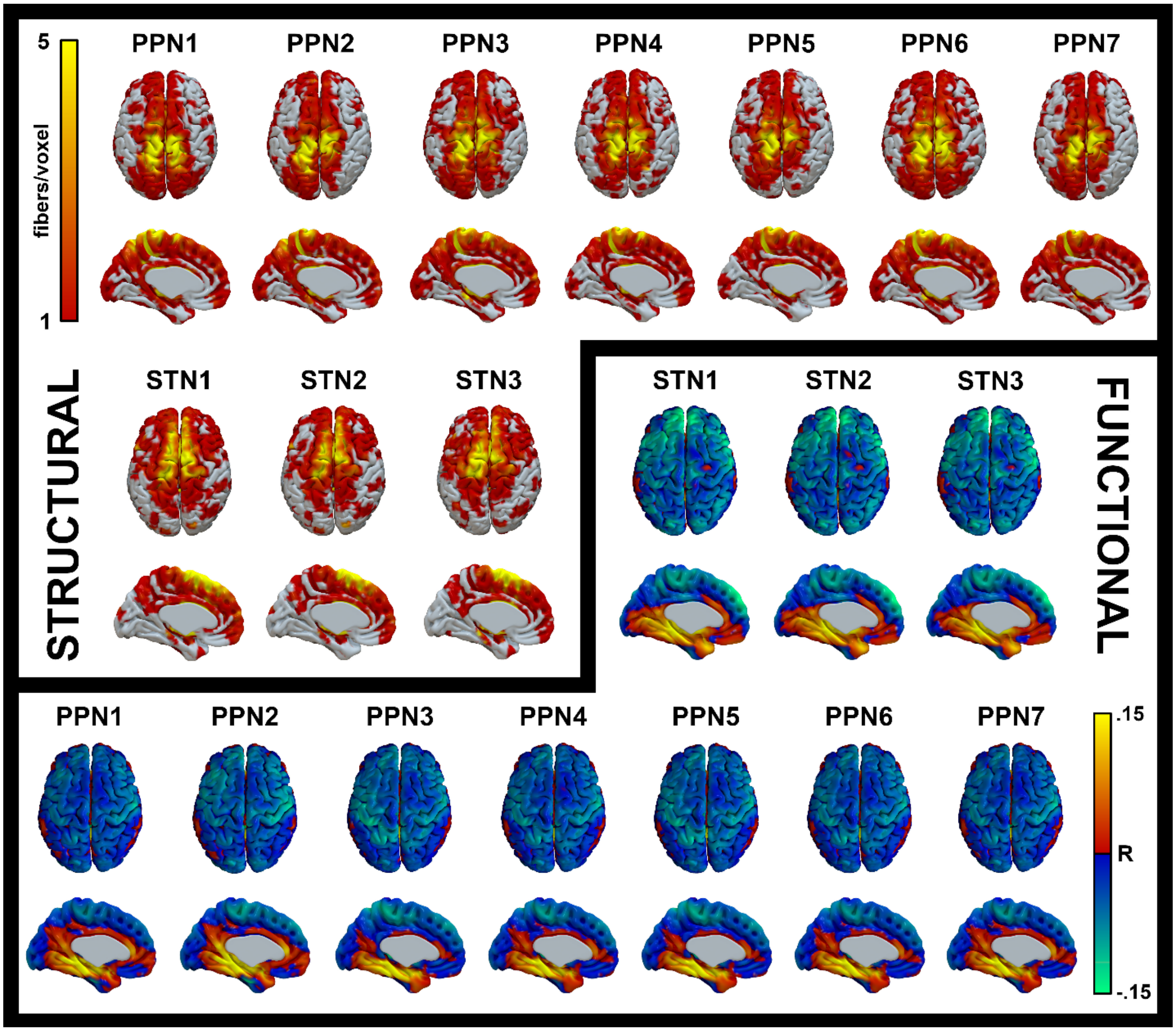

Supplementary Figure 1. Patient-wise structural and functional connectomic profiles. Activated fibers and fMRI correlates associated with individual patient PPNa-DBS VTA profiles are displayed.
